# Reprocessing of PLA/Graphene Nanoplatelets Nanocomposites

**DOI:** 10.3390/polym10010018

**Published:** 2017-12-24

**Authors:** Luigi Botta, Roberto Scaffaro, Fiorenza Sutera, Maria Chiara Mistretta

**Affiliations:** Department of Civil, Environmental, Aerospace and Materials Engineering, University of Palermo, RU INSTM of Palermo, Viale delle Scienze, 90128 Palermo, Italy; luigi.botta@unipa.it (L.B.); roberto.scaffaro@unipa.it (R.S.); fiorenza.sutera@unipa.it (F.S.)

**Keywords:** recycling, nanocomposites, graphene nanoplatelets (GnP), poly(lactic acid) (PLA)

## Abstract

This work reports a study on the effect of multiple reprocessing on the properties of poly(lactic acid) (PLA) filled with graphene nanoplatelets (GnP) compared to the melt reprocessed neat polymeric matrix. In particular, morphological, X-Ray Diffraction and Micro-Raman analyses, intrinsic viscosity measurements, thermal, rheological and mechanical tests were carried out on materials reprocessed up five times by means of a single screw extruder. The results indicated that the presence of GnP decreased the degradation rate as a function of the reprocessing cycles in comparison with the neat PLA that, on the contrary, showed a more drastic reduction of the molecular weight. Moreover, the reprocessing improved the particle dispersion and reduced the presence of GnP aggregates.

## 1. Introduction

Biopolymers are considered a sustainable alternative to oil-based synthetic polymers since they are renewable and do not contribute to environmental pollution being biodegradable, and therefore, they are currently used in several applications [1,2,3,4,5]. However, there are several valid reasons for recycling biopolymers, i.e., the growing industrial demands for biopolymers; recycling of biopolymers is crucial in reducing the consumption of renewable resources; the production and processing of biopolymers require a considerable amount of energy; some commercial biopolymers are not degraded under ordinary conditions; disposal of biopolymer articles has the disadvantage of discarding valuable raw materials [6,7]. Moreover, mechanical recycling allows for multiple lifecycles of a given plastic and thus substitutes and saves virgin material. Nevertheless, some preconditions for establishing mechanical recycling of bioplastics are necessary, such as: (i) the amount of bioplastics in the recycling stream is growing to a level that justifies additional investments by the recycling industry or (ii) a market demand for the specific recycled polymer.

Among biopolymers, poly(lactic acid) (PLA) gained much attention because of its interesting properties including good processability, mechanical properties and performance, making it a good candidate for replacing traditional polymers in several applications and furthermore, suitable for mechanical recycling [7].

Several papers investigated the possibility to recycle biodegradable polymers in order to reduce the environmental impact related to the life cycle of biodegradable polymer-based items [7,8,9,10,11,12,13]. In particular, a recent review focused the attention on the mechanical recycling of PLA and discussed the different valorization techniques that can be combined to optimize the value of PLA goods along its life-cycle [7].

However, a large use of biopolymers is often limited by the need of improving some functional properties such as mechanical and barrier properties. Therefore, intense efforts have been made to improve their physical properties in order to enhance the commercial potential of biopolymers such as PLA [14,15,16,17,18]. An effective way used to improve the properties of biopolymers was to incorporate nano-sized reinforcements in the matrix [19,20,21,22,23]. Indeed, it is well known that nanocomposites, i.e., polymers filled with particles having at least one dimension in the nanoscale range, show unique properties because of the peculiar increase of the matrix-filler interface [24]. In particular, graphitic nanofillers have extensively been used to prepare nanocomposites based on both conventional petroleum based plastics and bio-based polymers [25,26,27,28,29,30]. Among them, graphene or graphite nanoplatelets (GnP), also called graphite nanosheets or nanoflakes, are a relatively new carbon nanomaterial composed of stacked 2D graphene sheets with outstanding electrical, thermal and mechanical properties [25]. Moreover, studies concerning biocompatibility of graphene and graphene-based materials were reported demonstrating that they are non-toxic for human osteoblasts and mesenchymal stromal cells [31] and have good biocompatibility promoting cell viability and cell proliferation [32], although the biocompatibility can be size-dependent [33,34]. Furthermore, adding GnP to a biopolymeric matrix can slow down the decay of its mechanical performance during biodegradation [35].

Recently, some works studied the effect of reprocessing of the nanocomposites based on biopolymer matrices [36,37,38]. Tesfaye et al. investigated the effect of silk nanocrystals (SNCs) on the thermal and rheological properties of PLA under repetitive extrusion process. They showed that the presence of SNCs facilitated the crystallization process and delayed the thermal degradation of PLA matrix [38].

Peinado et al. studied the effect of extrusion on the mechanical and rheological properties of PLA reinforced with silicate nanoclays [37]. They concluded that despite the fact that both PLA and reinforced PLA materials showed a decrease in the viscosity during each reprocessing step, no remarkable loss in their mechanical properties was observed.

In our previous work, the properties of melt reprocessed PLA/hydrotalcites nanocomposites were studied up five subsequent extrusion cycles [36]. The results showed that reprocessing caused a remarkable decrease of viscous molar mass especially in the nanocomposites. Both the hydrotalcites—organically modified and unmodified—caused the increase of the thermo-mechanical degradation rate of the matrix.

The aim of this work was to evaluate the effect of reprocessing on the properties of PLA filled with graphene nanoplatelets (GnP) compared to the neat polymeric matrix. In particular, morphological analyses, intrinsic viscosity measurements, thermal, rheological and mechanical tests were carried out on materials reprocessed by means of five subsequent extrusion cycles.

## 2. Materials and Methods

### 2.1. Materials

The polymer matrix used in this work was a sample of a PLA (Ingeo™ Biopolymer 4032D) supplied by NatureWorks, Minnetonka, MN, USA. It is an extrusion grade with a melt flow rate (MFR) of 7 g/10 min (210 °C, 2.16 kg), density of 1.24 g/cm^3^ and melting point of 155–170 °C.

Graphene nanoplatelets (GnP), trade name xGnP^®^, Grade C, were supplied by XG Sciences Inc., Lansing, MI, USA. According to the manufacturer GnP used in this work have the following characteristics: average thickness lower than 2 nm; average diameter between 1 and 2 µm and a specific surface area of about 750 m^2^/g. Production of this GnP grade is based on exfoliation of sulphuric acid-based intercalated graphite by rapid microwave heating, followed by ultrasonic treatment [39].

### 2.2. Preparation of Nanocomposites and Reprocessing

Nanocomposites (containing 5 wt % of filler) were prepared by a co-rotating twin-screw extruder (type EBV 19/35 D, OMC, Saronno, Italy). Mechanically mixed polymer pellets and GnP were feeded at a rotational speed of 16 rpm and processed at a screw rotational speed of 220 rpm. The extruder temperature profile adopted was 180–190–190–200–200–200–190 °C. For comparison, neat PLA was processed under the same conditions adopted for the nanocomposites. Then, the obtained materials were pelletized for further characterization and/or reprocessing.

In order to prevent possible hydrolytic scission of the matrix during processing, both PLA and GnP were preventively dried overnight under vacuum at 90 °C and 110 °C, respectively.

Specimens for further characterizations were prepared by compression molding at 190 °C and 100 bar using a laboratory press (Carver, Wabash, MN, USA).

Reprocessing was carried out using a single screw extruder (Thermo Scientific HAAKE PolyLab QC, Karlsruhe, Germany) for five subsequent extrusion cycles. The temperature profile was set to 180–190–200–190 °C while the screw rotational speed was 50 rpm. For comparison, reprocessing was carried out both on PLA nanocomposites and on neat PLA under the same conditions. Before each step, the materials were dried overnight under vacuum at 90 °C, in order to prevent phenomena of hydrolytic chain scission. The molten material coming out from the extruder die was cooled in air and afterwards pelletized to be used for further characterizations. After each further extrusion process, an amount of the material was kept for analysis. The sample codes, the compositions and the reprocessing step of all the investigated materials are reported in Table 1.

### 2.3. Characterizations

The morphology of all the materials, including neat GnP, was analyzed by scanning electron microscopy (SEM; Quanta 200 ESEM, FEI, Hillsboro, OR, USA). In particular, the GnP powder was directly glued onto a sample holder whereas the polymeric samples were fractured under liquid nitrogen and then glued onto a sample holder. All the samples were sputter coated with a thin layer of gold under argon atmosphere for 120 s (Scancoat Six Edwards, Crawley, UK) in order to avoid electrostatic charging under the electron beam.

X-Ray Diffraction (XRD) analysis was performed by an X-ray diffractometer (RIGAKU model: D-MAX 25600 HK, Rigaku, Tokyo, Japan). Diffraction patterns were obtained in the 2θ range from 10° to 50° with a sampling width of 0.004° and a scan speed of 4°/min, using Cu Kα radiation (λ = 1.54 Å).

Micro-Raman analysis was performed at room temperature through a Renishaw InVia Raman Microscope spectrometer equipped with a 532 nm Nd:YAG laser excitation. Measurements were carried out in the range 3000–500 cm^−1^ with a spectral resolution of 0.5 cm^−1^.

The rheological characterization was performed using a plate-plate rotational rheometer (HAAKE MARS, Thermo Scientific, Waltham, MA, USA), operating at 190 °C on samples obtained by compression molding as above described. The instrument has been set to operate in the frequency sweep mode in the range 0.1–500 rad/s with a strain of 5%. Before testing, the samples were dried overnight under vacuum at 90 °C. The tests were performed in triplicate.

Tensile mechanical measurements were carried out by using a Universal Testing Machine (Instron model 3365, Instron, High Wycombe, UK) on rectangular shaped specimens (10 × 90 mm) cut off from sheets (thickness about 0.5 mm) prepared by compression molding as above described. The grip distance was 30 mm and the crosshead speed was 5 mm/min. Eight samples for each material were tested.

Thermal properties of the processed materials were studied using a differential scanning calorimeter (Perkin Elmer DSC 7, Waltham, MA, USA). The experiments were performed under N_2_ gas flow (20 mL/min). Samples underwent a heating/cooling/heating program in the temperature range 30–200 °C. The heating rate was 10 °C/min and the cooling rate was 40 °C/min. The experiments were performed in triplicate.

The crystallinity (*χ*) of PLA and its nanocomposites was calculated by Equation (1):

(1) $$\chi =\frac{\Delta H_{m}-\Delta H_{cc}}{\Delta H_{m}^{0}}\;\times 100$$

where Δ*H_m_* and Δ*H_cc_* are, respectively, the melting enthalpy and the cold crystallization enthalpy of the sample; $\Delta H_{m}^{0}$ is the melting enthalpy of 100% crystalline PLA (93.7 J/g) [40].

Enthalpy values found for nanocomposites were normalized on the actual amount of polymer involved in the thermal transition, being GnP not involved in melting/crystallization processes.

The intrinsic viscosity [*η*] was measured by means of a iVisc Capillary Viscometer LMV 830 (Lauda Proline PV 15, Lauda-Königshofen, Germany) instrument equipped with a Ubbelohde (*K* = 0.009676) capillary viscometer in an oil bath thermostated at 35 °C.

In order to prepare the solution at the concentration of 0.2 wt %, each material was dissolved in THF under stirring at 50 °C for 1 h. Flow time measurements were performed in triplicate for each sample until the standard deviation was below 0.5 s.

The intrinsic viscosity values was calculated according to Solomom-Ciuta by Equation (2) [41]:

(2) $$\left[\eta \right]=\frac{\sqrt{2}}{c}\cdot \sqrt{\eta_{sp}-ln\eta_{rel}}$$

where *c* is the concentration of the polymer solution, [*η*], *η_sp_* and *η_rel_* are, respectively, intrinsic, specific and relative viscosity. The solution viscosity of each sample was obtained by averaging 5 flow measurements. The viscosimetric molecular weight (*M_v_*) was calculated using the Mark-Houwink’s equation (Equation (3)):

(3) $$\left[\eta \right]=KM_{v}^{a}$$

The parameter values of the Mark-Houwink constants, *a* and *K*, depend upon the specific polymer solvent system. For PLA-THF, *K* = 1.74 × 10^–4^ and *a* = 0.736 [42].

## 3. Results and Discussion

### 3.1. Characterization of Neat GnP Powder

The suitability of GnP as filler for polymeric composites depends on their characteristics such as morphology and structural defects that can play a crucial role in the final properties of these systems. For these reasons the pristine GnP powder was characterized by SEM, X-ray Diffraction and Micro-Raman analyses.

**Figure 1:**
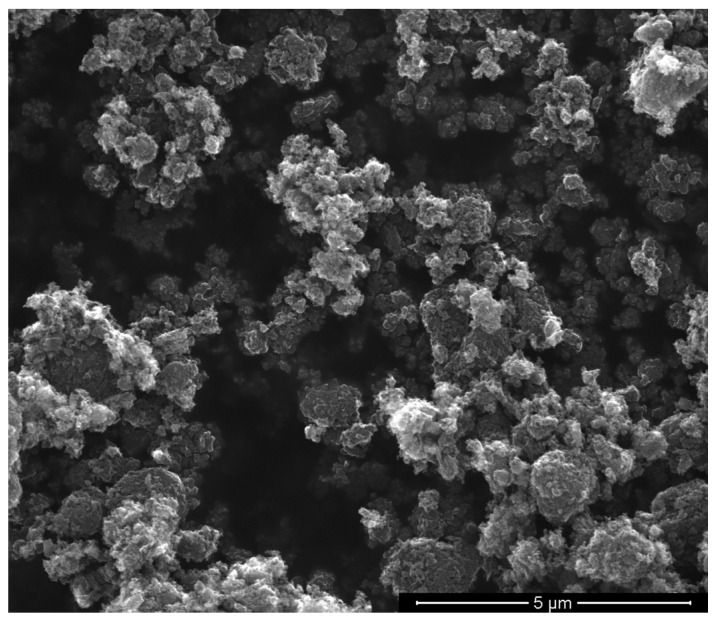
Scanning electron microscopy (SEM) micrograph of neat graphene nanoplatelets (GnP) powder.

The SEM micrograph of neat GnP powder (Figure 1) revealed that it tends to form irregular aggregates having different dimensions even larger than some micrometers.

**Figure 2:**
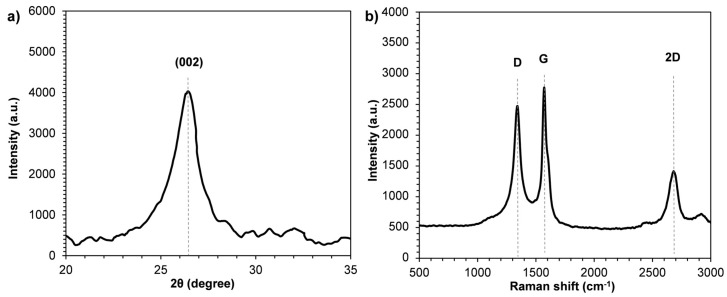
(**a**) X-Ray Diffraction (XRD) pattern and (**b**) Raman spectrum of neat GnP powder.

In Figure 2, XRD pattern and Raman spectrum of GnP are reported. In the GnP diffraction pattern (Figure 2a), the characteristic (002) diffraction peak appeared at around 2θ = 26.4°, indicating that the distance between graphitic layers is about 3.4 Å [43,44].

The Raman spectrum of GnP (Figure 2b) showed the typical two distinctive peaks called the D peak (located at 1340 cm^−1^) and the G peak (located at 1571 cm^−1^). As known, the D peak is due to the Raman scattering induced by zone-boundary phonons that reflects disordered structures. Such disordered structures include defects, edges, crystal boundaries, symmetry breaking, etc. The G peak is due to the stretching motion of sp2 bonds between carbon atoms, which reflects the crystalline graphite structure of graphite/graphene materials. Moreover, the Raman spectrum of GnP showed a peak located at 2680 cm^−1^, called 2D peak. It reflects the stacking structure of graphite along the *c*-axis, and it is well known to be very sensitive to the number of graphene layers in a flake [45].

### 3.2. Scanning Electron Microscopy (SEM)

**Figure 3:**
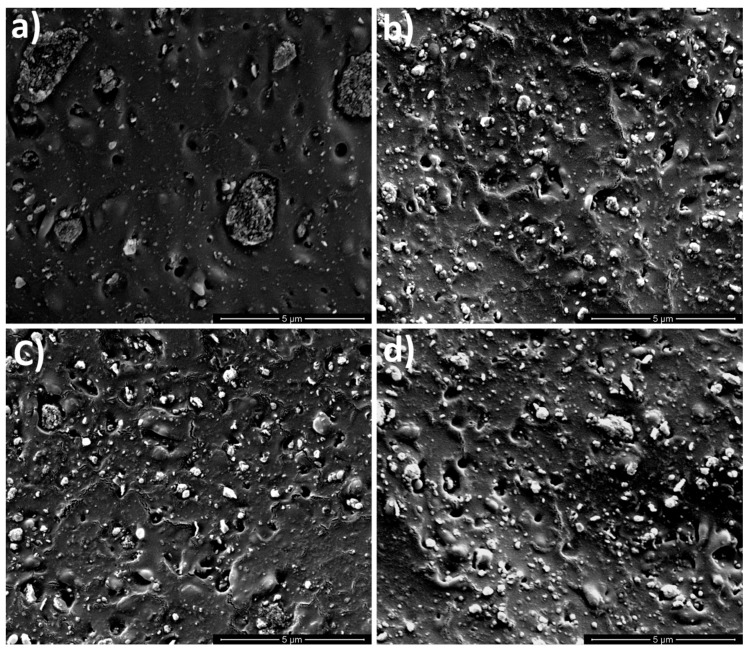
SEM micrograph of (**a**) poly(lactic acid) (PLA) + GnP R0; (**b**) PLA + GnP R1; (**c**) PLA + GnP R3; (**d**) PLA+ GnP R5.

The characteristics of multiphase systems such as nanocomposites certainly depend on the nature of the components but also on the final morphology achieved through the compounding. The SEM micrographs of the nanocomposite systems reprocessed up five times are reported in Figure 3. The nanocomposite obtained with the twin screw extruder, i.e., the not reprocessed one (PLA + GnP R0) showed a poor dispersion, as larger aggregates of GnP with dimensions similar to those of neat GnP are well visible (Figure 3a). Moreover, the adhesion between the particles and the matrix is quite poor as revealed by the presence of some voids around the GnP. On the contrary, the reprocessed samples, even that reprocessed one time, (Figure 3b–d) showed a higher level of particle dispersion and a lower presence of aggregates if compared with PLA + GnP R0 (Figure 3a). This improvement can be attributed to longer residence time of the material in the extruder during the reprocessing in which high levels of strain applied to the melt can promote enhanced mixing of the dispersed phase as already reported for similar systems [36,46,47]. However, the polymeric matrix revealed a more damaged morphology on increasing the reprocessing cycles and the adhesion between the matrix and the filler appeared to be slightly worse with the presence of more holes around the GnP particles.

### 3.3. X-ray Diffraction (XRD) Analysis

**Figure 4:**
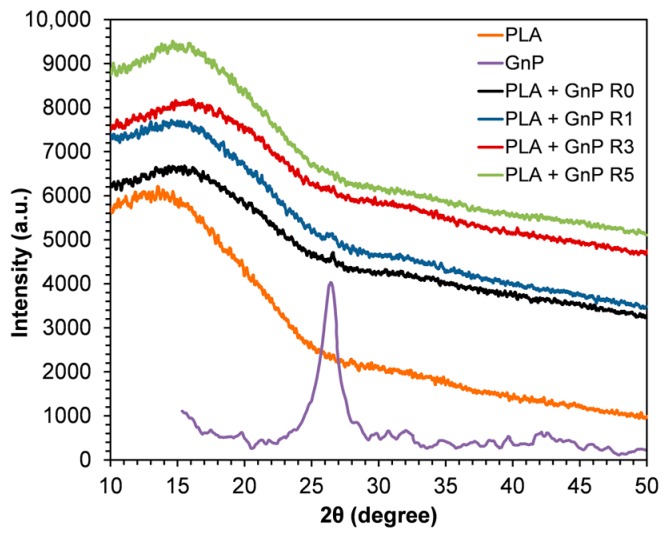
XRD patterns of neat GnP, PLA R0 and PLA + GnP nanocomposites at different reprocessing cycles.

To further investigate the structural features of nanocomposites, XRD patterns of the nanocomposite systems reprocessed up five times were obtained and are reported together with XRD patterns of neat GnP and PLA R0 in Figure 4. A broad amorphous peak was observed in neat PLA indicating that neat PLA had predominantly an amorphous microstructure. A small peak around 26.4° which corresponds to the characteristic peak of GnP appeared in the pattern of the not reprocessed nanocomposite (PLA + GnP R0), showing that the graphene layer is unable to disperse or completely separate and some sheets are still present in stacks form [43]. On increasing the reprocessing cycles, the peak related to GnP became less intense and disappeared in PLA + GnP R5. These results could be attributed to the lower amount of ordered layer structure of GnP, i.e., the disappearance of peak could be due to the exfoliation and random distribution of the platelets within the polymer matrix caused by reprocessing [43].

### 3.4. Micro-Raman Analysis

**Figure 5:**
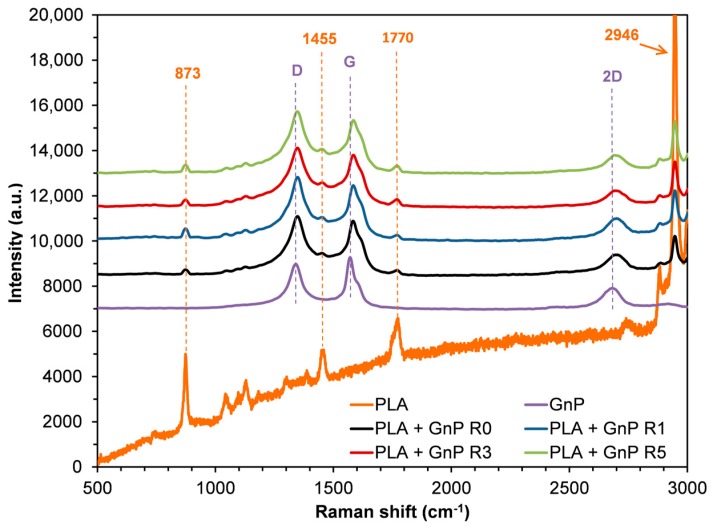
Raman spectra of neat GnP, PLA R0 and PLA + GnP nanocomposites at different reprocessing cycles.

In Figure 5, the Raman spectra of the nanocomposite systems reprocessed up five times together with spectra of neat GnP and PLA R0 are reported. The Raman spectrum of PLA exhibited characteristic bands at 873, 1455, 1770 and 2946 cm^−1^. The prominent band at 2946 cm^−1^ is assigned to the CH_3_ symmetric stretch. The peaks at 873 and 1455 cm^−1^ are the νC–COO and δCH3 asymmetric modes, respectively while the band located at 1771 cm^−1^ is assigned to C=O stretching [48,49,50]. These characteristic peaks were well visible in the PLA based nanocomposites together with the distinctive peaks of GnP, i.e., D, G and 2D bands. It is worth noting that no shift of bands related to PLA occurred in the nanocomposites. On the contrary, in PLA + GnP systems D and 2D peaks slightly shifted to higher wavenumbers. Moreover, G band exhibited a shift from 1571 cm^−1^ (neat GnP) to 1583, 1584 and 1585 cm^−1^ for PLA + GnP R0, PLA + GnP R1 and PLA + GnP R3/R5, respectively. The spectral blue-shifts could be ascribed to the disturbing of the graphene structure caused by the stresses acting on GnP, occurred during the processing operations [32]. Moreover, the shift of G band to higher wavenumbers could be attributed to reduction in number of graphene layers, corroborating the results of XRD analyses [51].

**Figure 6:**
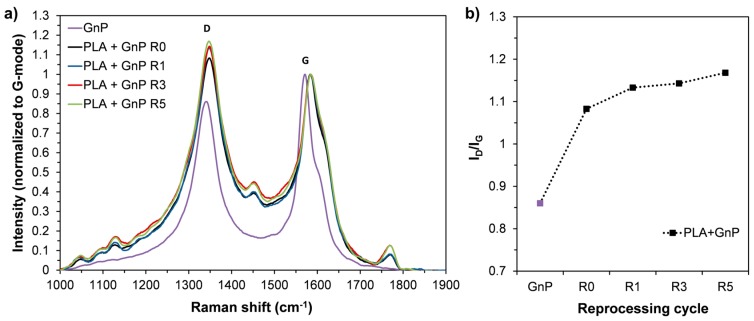
(**a**) Raman spectra normalized to G-mode of neat GnP and PLA + GnP nanocomposites at different reprocessing cycles; (**b**) I_D_/I_G_ as a function of reprocessing cycles for neat GnP and PLA + GnP systems.

In Figure 6 Raman spectra normalized to G-mode (Figure 6a) are reported together with I_D_/I_G_ as a function of reprocessing cycles (Figure 6b). The intensity ratio of the D to G-bands (*I*_D_/*I*_G_) is generally considered a measure of the degree of disorder, i.e., the larger the ratio the more defects present. The I_D_/I_G_ ratio of GnP increased after the incorporation in PLA matrix, i.e., from 0.86 for neat GnP to 1.08 for PLA + GnP R0. Moreover, the I_D_/I_G_ ratio further increased on increasing the reprocessing steps, suggesting that extrusion and reprocessing operations introduced structural defects into GnP [32]. However, the increase of I_D_/I_G_ could also be attributed to GnP disaggregation/exfoliation and therefore, more carbon surface interaction with the matrix [51].

### 3.5. Rheological Properties

**Figure 7:**
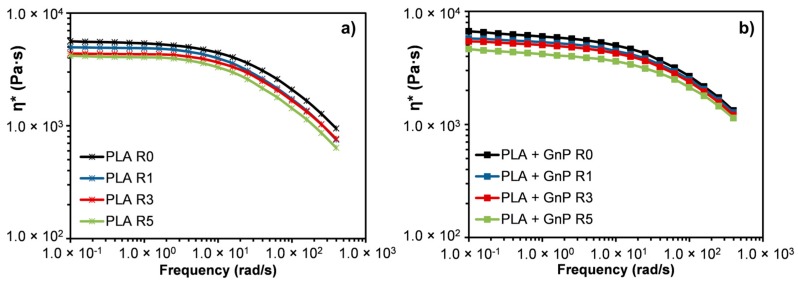
Complex viscosity as a function of frequency of (**a**) PLA and (**b**) PLA + GnP underwent to reprocessing.

The complex viscosity as a function of frequency was reported in Figure 7 for all the systems presented in this work. At low frequencies, neat PLA exhibited a Newtonian plateau regardless of the extrusion number. Moreover, the viscosity of PLA decreased on increasing the extrusion cycles likely due to polymer chain degradation. Indeed, three possible hypotheses can be assumed for explaining degradation phenomena of the matrix occurring during processing: (a) radical degradation; (b) hydrolysis and/or (c) transesterification with residual catalysts [52].

PLA + GnP nanocomposites (Figure 7b) showed the same trend as PLA as a function of reprocessing cycles. However, the viscosity of the samples filled with GnP was slightly higher than that of neat PLA, and the flow curve exhibited a slightly more pronounced non-Newtonian behavior at low frequencies if compared with the unfilled polymer. This rheological behavior is reported as a typical behavior shown by several nanocomposite systems including polymer/clay nanocomposites [53,54] and polymer/GnP nanocomposites [28,29,55,56]. It is generally attributed to an interaction between the dispersed filler and the matrix that restricts the polymer chain movements. Moreover, the drop of the viscosity at highest frequencies for the filled systems was more slight if compared with the neat PLA. This behavior can be likely attributed to the presence of GnP hindering probably further degradation phenomena during the rheological test. It is worth noting that, for the filled systems, two opposite phenomena occurred during the reprocessing, i.e., the subsequent extrusion led to degradation of the polymer matrix but improved, at least after the first reprocessing, the filler dispersion.

**Figure 8:**
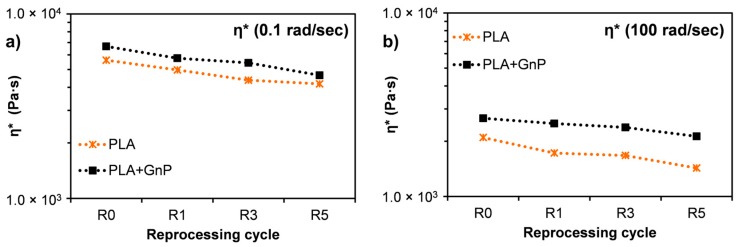
Complex viscosity as a function of reprocessing cycles for PLA and PLA + GnP systems at (**a**) 0.1 rad/s and (**b**) 100 rad/s.

The comparison between the rheological behavior of reprocessed PLA and reprocessed PLA + GnP was more visible in Figure 8 where the viscosity at 0.1 rad/s and 100 rad/s as a function of the reprocessing cycle was reported. In particular, it is evident that, although for both the systems the viscosity decreased upon the reprocessing cycles, the filled system showed an always higher viscosity than that of neat PLA. Moreover, it is evident that the differences between the viscosity of PLA and the filled systems is larger at higher frequency.

### 3.6. Intrinsic Viscosity

**Figure 9:**
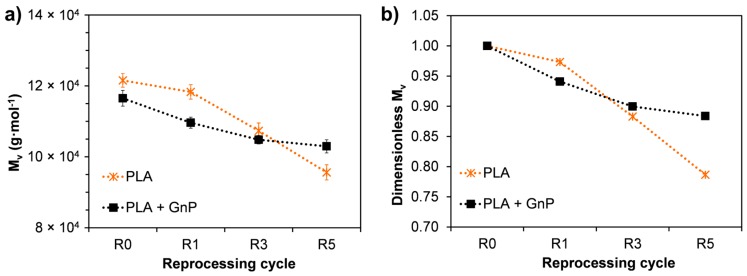
Viscous molar mass (*M_v_*) as a function of reprocessing cycles (**a**) and dimensionless *M_v_* as a function of reprocessing cycles (**b**) for PLA and PLA + GnP systems. Dimensionless values have been calculated as ratio between *M_v_* values of reprocessed materials and the *M_v_* value of the corresponding R0 material.

In order to verify the decrease of molecular weight due to degradation phenomena, intrinsic viscosity measurements were performed (Figure 9). Viscous molar mass (*M_v_*) as a function of reprocessing cycles is shown in Figure 9a. As expected, the results showed that the molecular weight of PLA decreased on increasing the reprocessing cycles reducing up to 22% at the fifth recycling. In particular, a more drastic reduction occurred from the third reprocessing cycle as more evident in Figure 9b, where the dimensionless values of *M_v_* are plotted. Indeed, the rate of decrease of *M_v_* of PLA clearly increased from the third recycling. On the contrary, the presence of GnP clearly reduced the rate of degradation as a function of the reprocessing in comparison with the neat PLA. This behavior can be probably attributed to the stabilizing effect of GnP. Indeed, several mechanisms have been proposed to explain the contribution of GnP in improving the thermal stability of polymer based nanocomposites such as the following: (i) GnP can act as “efficient heat sinks”, extracting more heat than the matrix and not allowing the accumulation of heat within the latter, thereby preventing oxidation at the early stages of degradation [25]; (ii) GnP could serve as mass transfer barriers (shielding effect) against the volatile pyrolized products [25]; (iii) GnP can create a tortuous path for air, delaying the thermo-oxidative degradation of the material [57].

### 3.7. Thermal Properties

Results derived from DSC measurements are summarized in Table 2. The glass transition temperature (*T*_g_) of neat PLA and nanocomposites were very close. Reprocessing did not influence the T_g_ of both the systems, which were in the narrow ranges of 60.1–60.7 °C. Cold crystallization was observed for both neat PLA and PLA + GnP samples. In particular, a progressive decrease of the cold crystallization temperature (*T*_cc_) was shown on increasing the extrusion number, in agreement with the results obtained for similar systems [8,9]. However, the decrement of *T*_cc_ as a function of the reprocessing cycles was more remarkable for PLA + GnP. This result can be likely attributed to the better dispersion of GnP underwent under reprocessing that can promote an easier cold-crystallization, as reported elsewhere for similar systems [36].

PLA matrix showed two melting peaks with the dominant peak at the higher temperature. Other works reported similar melting behavior for PLA and its nanocomposites [14,40,58]. The double peak was explained in different ways: (a) formation of a disordered alpha phase of PLA owing to the low crystallization temperature; (b) nucleation of more than one crystal structure and (c) different morphology of the lamellae formed prior to the heating process. On the contrary, in PLA + GnP samples bimodal distribution was not present, as already reported for similar systems [36].

The crystallinity of PLA slightly increased on increasing the reprocessing cycles since polymer chains shortening made easier crystallization kinetics. The increment of crystallinity as a function of reprocessing cycles was larger for the filled materials because of the synergistic effect played by GnP as nucleating agent together with the reduction of the polymer chains. Moreover, as above stated, the improved dispersion of GnP due to reprocessing, further increased the nucleating role of the filler. However, the influence of GnP on PLA crystallinity was quite modest as already reported by other authors for similar systems [59].

### 3.8. Mechanical Properties

**Figure 10:**
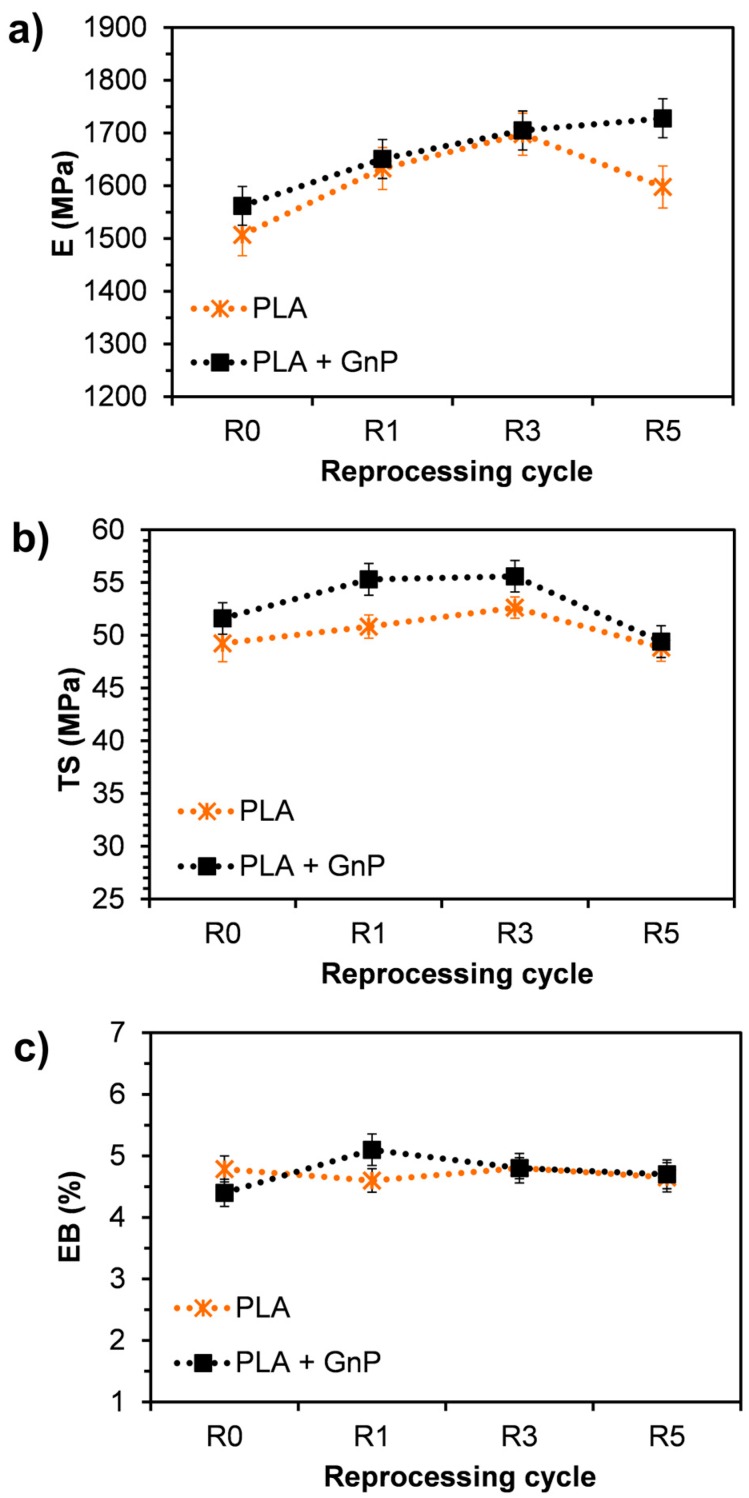
Mechanical properties of neat PLA and PLA + GnP as a function of reprocessing cycles: (**a**) Elastic modulus (E); (**b**) tensile strength (TS) and (**c**) elongation at break (EB).

The effects of reprocessing on the mechanical properties were displayed in Figure 10. Elastic modulus (E), tensile strength (TS) and elongation at break (EB) of the nanocomposites were compared with neat PLA.

The not reprocessed nanocomposites showed an elastic modulus slightly higher than that of the neat matrix processed under the same conditions (Figure 10a). Such a low increase in stiffness can be probably attributed to the quite poor morphology achieved through the compounding, i.e., the presence of large aggregates and a quite poor adhesion between the filler and the matrix, as shown above by SEM analysis. Moreover, although the presence of GnP reduced the rate of degradation as a function of the reprocessing in comparison with the neat PLA, the molecular weight of PLA + GnP R0 was lower than that of PLA R0. During the subsequent extrusion cycles, the elastic modulus of neat PLA increased up to the third recycling, afterwards decreased. These results can likely be attributed to the competition between two phenomena that occur during reprocessing, i.e., the decrease of molecular weight and the increase of crystallinity that affect in an opposite way the stiffness of the material [36,52]. On the contrary, the stiffness of the PLA filled with GnP increased as a function of the reprocessing cycles for all the extrusion steps. This behavior can be explained considering that, although also the reprocessing of this system led to the decrease of molecular weight of PLA, the presence of GnP reduced the rate of degradation. Furthermore, at the same time the reprocessing improved the morphology of the nanocomposite at least as regards the reduction of particle size and the increase of dispersion. Moreover, similarly to the neat PLA, reprocessing caused a slight higher increase of the polymer crystallinity.

PLA and PLA + GnP showed a similar trend of TS as a function of reprocessing cycles (Figure 10b). In particular, the values remained almost constant during the extrusion cycles, although exhibiting a maximum at the third reprocessing. However, the nanocomposites exhibited a TS slightly higher than that of the PLA for all the re-extrusion cycles.

The addition of GnP reduced very slightly the EB of the not reprocessed nanocomposite despite the presence of some aggregates and PLA + GnP R1 exhibited even an EB slightly higher than that of PLA R1 (Figure 10c). However, it was worth noting that even the not reprocessed PLA exhibited a brittle behavior, i.e., an elongation at break very low. Similar results were reported by other authors that attributed the preservation of EB to the small size of GnP used in this work [59]. Further reprocessing cycles did not significantly influence the EB both of PLA and of the nanocomposite system that has shown values very close to those of the neat matrix.

## 4. Conclusions

The effect of multiple reprocessing cycles on the properties of PLA/GnP nanocomposites and, for comparison, of neat PLA, was evaluated through morphological, XRD and Micro-Raman analyses, intrinsic viscosity measurements, thermal, rheological and mechanical tests. The nanocomposites were prepared by a co-rotating twin-screw extruder and reprocessed up five times by means of a single screw extruder.

The results indicated that the re-extruded nanocomposite samples, even that reprocessed one time, showed a better morphology, i.e., a higher level of particle dispersion and a lower presence of aggregates. XRD and Raman analyses suggested that the extrusion process and reprocessing led to a reduction in the number of graphene layers of GnP. The multiple reprocessing caused the decrease of viscosity and of the molecular weight on increasing the re-extrusion cycles both for neat PLA and for PLA based nanocomposites. However, the presence of GnP decreased the degradation rate as a function of the reprocessing cycles in comparison with the neat PLA that, on the contrary, showed a more drastic reduction of the molecular weight. Moreover, the presence of GnP led to an increase of PLA crystallinity as a function of the reprocessing cycles.

The stabilizing effect of GnP suggests that it could be used to increase the reprocessability of PLA without compromising other required properties of the matrix.

## Figures and Tables

**Table 1:** Composition of samples and their codes.

Sample Code	PLA (*w*/*w* %)	GnP (*w*/*w* %)	Processing
PLA R0	100	-	Extrusion
PLA R1	100	-	1st Reprocessing
PLA R3	100	-	3th Reprocessing
PLA R5	100	-	5th Reprocessing
PLA + GnP R0	95	5	Extrusion
PLA + GnP R1	95	5	1st Reprocessing
PLA + GnP R3	95	5	3th Reprocessing
PLA + GnP R5	95	5	5th Reprocessing

**Table 2:** Differential scanning calorimeter (DSC) results of PLA and PLA + GnP at different reprocessing cycles.

Sample	T_g_ (°C)	T_cc_ (°C)	*T*_m1_ (°C)	T_m2_ (°C)	Δ*H_cc_* (J/g)	Δ*H_m_* (J/g)	*χ* (%)
PLA R0	60.2 ± 0.1	110.9 ± 0.3	161.9 ± 0.1	167.9 ± 0.2	29.2 ± 0.1	32.6 ± 0.2	3.6 ± 0.1
PLA R1	60.5 ± 0.2	109.7 ± 0.2	161.5 ± 0.2	168.2 ± 0.3	29.1 ± 0.2	33.0 ± 0.1	4.1 ± 0.1
PLA R3	60.5 ± 0.1	107.4 ± 0.2	160.7 ± 0.2	168.2 ± 0.2	27.8 ± 0.3	32.0 ± 0.2	4.5 ± 0.2
PLA R5	60.2 ± 0.2	106.2 ± 0.3	160.2 ± 0.1	167.7 ± 0.2	27.6 ± 0.2	32.1 ± 0.1	4.8 ± 0.1
PLA + GnP R0	60.5 ± 0.1	111.5 ± 0.2	-	168.9 ± 0.3	31.1 ± 0.3	35.1 ± 0.3	4.2 ± 0.2
PLA + GnP R1	60.7 ± 0.2	101.7 ± 0.1	-	169.2 ± 0.1	29.5 ± 0.2	34.1 ± 0.2	4.9 ± 0.1
PLA + GnP R3	60.3 ± 0.2	99.3 ± 0.2	-	169.2 ± 0.2	27.5 ± 0.2	33.1 ± 0.1	6.0 ± 0.1
PLA + GnP R5	60.1 ± 0.1	98.0 ± 0.2	-	169.0 ± 0.2	26.2 ± 0.1	34.3 ± 0.2	8.6 ± 0.1
